# Electrically Conductive Hydrogels for Articular Cartilage Tissue Engineering

**DOI:** 10.3390/gels8110710

**Published:** 2022-11-03

**Authors:** Filipe Miguel, Frederico Barbosa, Frederico Castelo Ferreira, João Carlos Silva

**Affiliations:** 1 iBB—Institute for Bioengineering and Biosciences and Department of Bioengineering, Instituto Superior Técnico, Universidade de Lisboa, Av. Rovisco Pais, 1049-001 Lisboa, Portugal; 2 Associate Laboratory i4HB—Institute for Health and Bioeconomy, Instituto Superior Técnico, Universidade de Lisboa, Av. Rovisco Pais, 1049-001 Lisboa, Portugal

**Keywords:** articular cartilage, conductive materials, electrical stimulation, hydrogels, nanocomposites, tissue engineering

## Abstract

Articular cartilage is a highly specialized tissue found in diarthrodial joints, which is crucial for healthy articular motion. Despite its importance, articular cartilage has limited regenerative capacities, and the degeneration of this tissue is a leading cause of disability worldwide, with hundreds of millions of people affected. As current treatment options for cartilage degeneration remain ineffective, tissue engineering has emerged as an exciting approach to create cartilage substitutes. In particular, hydrogels seem to be suitable candidates for this purpose due to their biocompatibility and high customizability, being able to be tailored to fit the biophysical properties of native cartilage. Furthermore, these hydrogel matrices can be combined with conductive materials in order to simulate the natural electrochemical properties of articular cartilage. In this review, we highlight the most common conductive materials combined with hydrogels and their diverse applications, and then present the current state of research on the development of electrically conductive hydrogels for cartilage tissue engineering. Finally, the main challenges and future perspectives for the application of electrically conductive hydrogels on articular cartilage repair strategies are also discussed.

## 1. Introduction

Articular cartilage (AC) is a highly complex and specialized connective tissue present in diarthrodial joints and is paramount for joint mobility and health. Regarding cell populations, AC is composed uniquely of chondrocytes, which represent about 2% of the tissue’s volume, and play a crucial role in its development, maintenance, and production of extracellular matrix (ECM) [1]. While chondrocytes rarely perform direct signal transduction through cell-to-cell contacts, their phenotype is responsive and dependent on biophysical stimuli such as mechanical, electrical, and magnetic stimuli [2,3]. These cells are embedded in a dense ECM, composed primarily by type II collagen, which forms fibrils that intertwine with aggregating proteoglycans. The latter are heavily glycosylated proteins consisting of a protein core with covalently attached glycosaminoglycans (GAGs) chains. Chondroitin sulfate is the most abundant proteoglycan in AC by weight and is able to interact with hyaluronic acid (HA) to form large aggregates of proteoglycans [4]. The presence of collagen and proteoglycans within the ECM leads to the swelling of AC with water, the most abundant component of the tissue, comprising 80% of its wet weight. The water flow through the cartilage aids in the transport of nutrients to chondrocytes, while also providing lubrication. Different sets of phospholipids (e.g., phosphatidylcholine) adsorbed onto the surface of AC are also responsible for the tissue’s low friction coefficient, by promoting the quick breaking and reestablishment of weak van der Waals bonds between hydrophobic acyl tails on opposing cartilage surfaces [5,6].

AC has a complex, multilayered structure, with each zone having different densities of chondrocytes, ECM composition and organization, and water content. The superficial zone makes up to 10–20% of total AC thickness, with collagen fibers aligned in parallel with the articular surface and a relative high number of flattened chondrocytes. Around 40 to 60% of the AC volume corresponds to the middle zone, containing a lower density of spherical chondrocytes and collagen fibers organized obliquely. The deep zone represents 30% of AC thickness and contains the highest diameter collagen fibers in a radial disposition and proteoglycan density, with the lowest number of chondrocytes [7].

The intrafibrillar water present within the tissue is able to move through the ECM when the tissue experiences compression, despite existing a high frictional resistance to this flow within the tissue. It is the combination of these mechanisms that grant AC its unique mechanical properties [8]. This tissue mainly functions as a smooth, lubricated surface at the end of bones in diarthrodial joints, allowing low friction articulation, while being capable of transmitting loads to the underlying bone. AC is able to withstand very high cyclic loads, sometimes several times an individual’s bodyweight, without significant damage or degeneration [9]. However, despite displaying a high resistance to damage when sustaining loads, AC is also characterized by severely limited intrinsic repair capabilities. Due to its low cell density and avascular nature, the tissue often lacks access to reparative cells and growth factors, impairing regeneration.

Osteoarthritis (OA) is the most common degenerative joint disorder, characterized by a progressive loss of AC and severe joint pain and stiffness [10]. In fact, it is a leading cause of disability, with an estimated 250 million people currently affected by OA, with knee OA affecting approximately 12% of men and 14% of women [11,12,13]. Furthermore, the number of people affected by OA is estimated to keep increasing as a consequence of the obesity epidemic and population aging, with data suggesting that an additional 26 thousand per 1 million adults will experience OA by 2032 [14]. Despite its incidence, the pathogenesis of OA remains elusive, and an effective treatment has not yet been achieved.

Pharmaceutical therapy is the most common option for OA treatment, and largely consists of acetaminophen, non-steroidal anti-inflammatory drugs, opioid analgesics, and serotonin–norepinephrine re-uptake inhibitors. However, these treatment options are primarily focused on pain relief and anti-inflammation, being unable to repair cartilage damage [15]. Other treatment options such as osteochondral autograft transplantation or autologous chondrocyte implantation (ACI) also carry various downsides that severely limit their use, with transplanted cartilage being more prone to damage, and the occurrence of donor site morbidity. The limited proliferation capacity and dedifferentiation of cultured chondrocytes have also impaired the success of the ACI technique [16].

Considering the unmet medical need for the effective treatment of OA, cartilage tissue engineering (CTE) has emerged as a promising alternative to treat AC defects [17]. CTE employs the use of a biocompatible, biodegradable, and biomimetic biomaterial scaffold that is combined with cells and bioactive factors (e.g., growth factors, physical stimuli) to promote cell proliferation, differentiation, and maturation, ultimately leading to tissue regeneration [18]. The complex architecture and limited self-repair potential of AC are two main challenges impairing successful CTE strategies. However, combining the fabrication of cell-seeded scaffolds, optimized to closely mimic the native tissue properties, with physiologically relevant biophysical/biochemical cues holds great promise for the effective repair of cartilage defects.

Concerning the main cell sources used in CTE strategies, mesenchymal stem/stromal cells (MSCs) have been widely explored as an alternative to chondrocytes due to their ready availability from different tissues (e.g., bone marrow, adipose tissue, muscle, periosteum, umbilical cord matrix, synovial membrane, and dental pulp), high in vitro expansion capacity, ability to differentiate towards cartilage upon proper induction factors, and their advantageous immunomodulatory/trophic properties [19,20]. MSCs from different sources have been demonstrated to have different chondrogenic differentiation potential, with some studies suggesting synovium-derived MSCs as a superior source for CTE strategies when compared to MSCs isolated from non-joint tissues [21,22,23]. Moreover, in recent years, alternative cell sources including articular cartilage progenitor cells (ACPCs) and induced pluripotent stem cells (iPSCs) have also been explored in CTE with highly promising results, particularly in terms of the lower hypertrophy of the generated cartilage tissues [24,25].

Various growth factors and other bioactive molecules have been used as candidates to promote cartilage regeneration. For example, TGF-β3 has been demonstrated to enhance the chondrogenesis of MSCs in vitro, through increased expression levels of chondrogenic markers [26]. Despite a wide array of other growth factors (FGF-2, BMP-7, BMP-2, IGF-1) [27], and even exciting new small molecules such as kartogenin [28] being successfully used to augment cartilage formation, some problems still emerge with the individual use of these bioactive factors. As tissue formation occurs over the course of weeks to months, a single delivery of these factors would not be sufficient to drive tissue repair, requiring continuous supplementation, which needs strictly optimized dosage and controlled delivery protocols. Furthermore, despite inducing chondrogenic differentiation, the uncontrolled delivery of some of the abovementioned growth factors can also trigger unwanted outcomes, such as ossification, synovial fibrosis, and even synovitis through intra-articular injections [29].

Hydrogels are highly absorbent polymer networks swollen in large quantities of water, yet maintaining well-defined structures [30]. Biomedical applications of hydrogels started about 60 years ago, and have since been successfully applied as drug carrier systems, diagnostic devices, and chemically modified implants for regenerative medicine [31]. One of the most enticing properties of hydrogels is their biocompatibility, although many factors can influence the immune system response to their presence. Hydrogels are highly customizable, having an array of parameters such as polymer type, mechanical properties, porosity, degradation profile, cell inclusion and release of bioactive factors, that can be modified in order to tailor its properties and function specifically for CTE applications [32]. Hydrogel stiffness and strength can be modulated to mimic the native AC tissue, as this parameter is crucial to replicate its physical properties, but can also influence cellular processes such as cell adhesion and differentiation [33]. Ideally, a hydrogel for AC repair should recapitulate the biomechanical properties described for human healthy cartilage tissue (ranges of compressive modulus (0.24–0.85 MPa), elastic modulus (5–25 MPa), and tensile strength (15–35 MPa)) [34,35]. Porosity is another important customizable parameter as, just as in native AC, it dictates the flow of nutrients and solutes to the local cells, while enabling the removal of waste produced during cell metabolism. The optimal hydrogel porosity and pore size for cartilage regeneration is still a topic of debate, as some studies have found smaller pore sizes (50–150 μm) to induce chondrocyte dedifferentiation [36], while other works have observed an increased metabolic activity and cartilage-like ECM synthesis when compared to larger pores [37]. Biocompatibility is a key parameter for a successful hydrogel implementation. Since the hydrogel will be in constant contact with the surrounding tissue, it is important that it does not trigger any inflammatory or immune responses, while supporting chondrocyte adhesion, growth, and ECM synthesis. Moreover, as the prolonged presence of a biocompatible hydrogel can hinder the growth of new tissue, the biodegradability of such hydrogels is also a key parameter for a successful TE strategy. Ideally, the degradation rate of the implanted hydrogel should perfectly match the production rate of newly formed cartilage tissue [38,39].

Hydrogels for CTE strategies have been fabricated using both natural and synthetic polymers. Hydrogels produced from natural polymers (e.g., alginate, gelatin, collagen, hyaluronic acid, chondroitin sulfate, fibrin) have monomers similar to native cartilage ECM, making them naturally more biocompatible and bioactive than synthetic polymer hydrogels [e.g., poly(acrylic acid), poly(ethylene glycol), poly(ethylene oxide), poly(vinyl alcohol)], which lack bioadhesive sites. However, natural polymer hydrogels also present important limitations compared to the synthetic polymer ones, such as weak mechanical properties (highly relevant for the regeneration of AC tissue, which is under constant mechanical loading), batch-to-batch variability, and difficult processability and control over structural properties and degradation rate [38,39,40].

These extensive customizable features alongside their native-like swelling properties, have allowed the widespread use of hydrogels in CTE strategies [41]. Hydrogels can be designed to be structurally and mechanically similar to cartilage, allowing the entrapment of cells such as in the native tissue’s ECM. Their viscoelastic properties enable the effective transmission of mechanical loads to chondrocytes, which require these biophysical cues for survival and function [42]. Some types of hydrogels are particularly interesting for CTE purposes, as they can be flowable aqueous solutions, and thus easily injectable, matching any shape of cartilage defect with posterior polymerization. The gelation of the main injectable hydrogel systems used in CTE has been achieved through several physical and chemical crosslinking methods, which can be controlled in a variety of ways depending on the hydrogel properties [39]. Depending on the trigger method used, injectable hydrogels can be classified as photo-crosslinked [43], enzymatically crosslinked [44], Schiff-base crosslinked [45], Michael type addition-mediated [46], ionic-crosslinked [47], disulfide-crosslinked [48], temperature-sensitive [49,50], and pH-sensitive hydrogels [50,51]. The degree and type of crosslinking can influence many of the scaffold’s properties such as swelling capacity and elastic modulus [39]. Over the years, a variety of biomaterials have been successfully utilized to fabricate injectable polymeric hydrogels, including heparin [52,53], collagen [54,55], gelatin [56], alginate [57], poly (ethylene glycol) (PEG) [58,59], chondroitin sulfate [60], hyaluronic acid [61,62], and chitosan [63,64] (Table 1). The customization potential of hydrogels has been extensively explored; however, there is now a focus on combining these scaffolds with relevant chemical/biophysical stimuli to more closely recreate the native tissue’s environment. In fact, some of these hydrogels have been successfully combined with chondroinductive factors for CTE applications [65,66,67]. Injectable thermosensitive chitosan-based hydrogels loaded with kartogenin (a chondroinductive small molecule) showed a sustained drug release for 40 days. Moreover, the in vitro culture results demonstrated an enhanced chondrogenic differentiation of human adipose-derived MSCs treated with the kartogenin-loaded hydrogels, in comparison to the cells supplemented with the pure drug [67].

One of the abovementioned biophysical cues is electrical stimulation, which has been shown to increase the proliferation of chondrocytes and secretion of ECM molecules, accelerating the repair of cartilage defects in vivo [68]. Interestingly, MSCs exposed to electrical stimuli have shown an increase in the production of the chondrogenic markers type II collagen, aggrecan, and GAGs [69]. The combination of conductive hydrogels with electrical stimulation allows the creation of a stimuli-responsive scaffold, capable of on-demand manipulation of the microenvironment, constituting a dynamic and powerful tool for CTE. Thus, there have been intense efforts to find new and improved ways to confer electrical conductivity to hydrogels with the incorporation of diverse conductive materials taking the spotlight.

**Figure 1:**
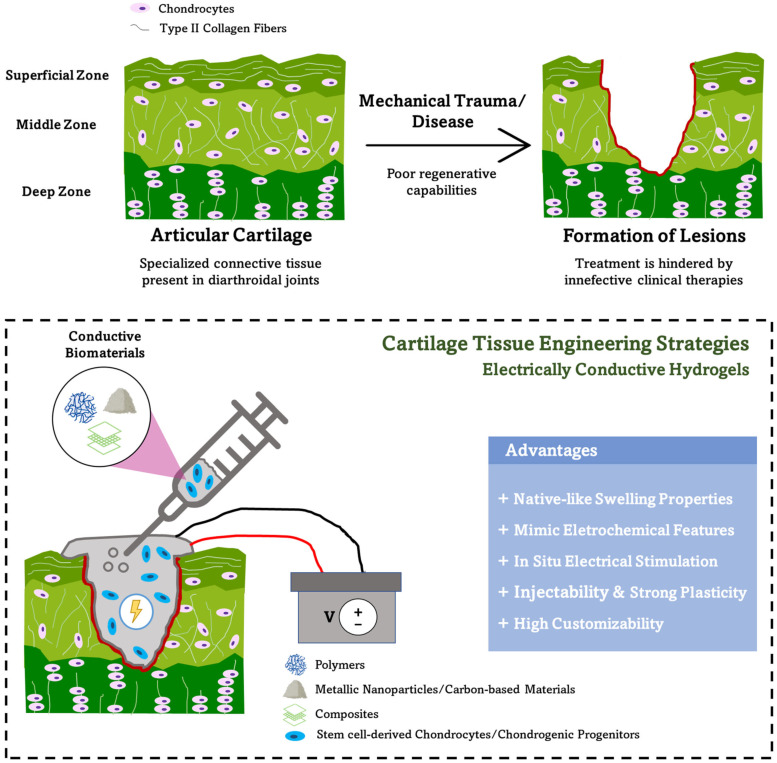
Electrically conductive hydrogels as a promising tool for the repair of articular cartilage defects caused by trauma or debilitating diseases such as osteoarthritis (top). These functional conductive hydrogels present several advantages for cartilage tissue engineering strategies (bottom).

In this review, a summary of the conductive biomaterials that have been utilized in conjunction with hydrogels is presented. The current state of development of electrically conductive hydrogels for AC regeneration strategies (Figure 1) is highlighted and discussed. Finally, some of the current challenges and possible future perspectives are also discussed.

## 2. Electrical Properties of Articular Cartilage Tissue

AC has intrinsic electrical/electrochemical properties, derived from the flow of free electrolytes (K^+^, Ca^2+^, Na^+^) through the fixed negative charges of carboxyl and sulfate groups attached to the GAGs in the side chains of proteoglycans [70]. The inhomogeneous distribution of fixed charges in the tissue also creates diffusion potentials, while fluid flow along the charged tissue results in a streaming potential. Local chondrocytes exposed to these electrical signals are responsive, being able to convert them into intracellular signaling. In this case, a signal transduction cascade ultimately leads to the production of SOX9, a transcription factor that triggers the synthesis of typical cartilage ECM components such as aggrecan and collagen type II [71,72]. With an increasing focus on finding ways to recreate the electrical properties of AC and the biophysical microenvironment found in native tissue, recent studies have brought to light the potential of external electric stimulation for CTE.

## 3. Conductive Materials for Tissue Engineering

A great variety of conductive materials can be implemented with hydrogels in order to create electrically conductive scaffolds. These platforms are invaluable for CTE as they can provide not only native-like physical properties, but are responsive to relevant biophysical cues such as electrical stimulation, simulating a more physiological environment. Despite some combinations of hydrogels and conductive materials not yet being directly applied for CTE, most of the ones presented have found uses on diverse regenerative medicine applications for the engineering of other tissues such as cardiac or neural. Through the study of their different applications, it is possible to further explore the potential of these materials for CTE.

### 3.1. Metallic Nanoparticles

Metallic nanoparticles are electrically conductive nanosized particles, with a metal core shelled by an inorganic or organic metal or metal oxide, behaving differently according to their size, shape, and type of material. It is possible to functionalize the surface of nanoparticles in order to strengthen the interaction with polymers [73]. Electrically conductive nanocomposite hydrogels have been produced with gold [74], silver [75], platinum [76], and metallic oxide (zinc oxide, ZnO) nanoparticles [77] (Table 2). ZnO is a semiconducting and piezoelectric material, which becomes conductive when highly n-type doped with aluminum or gallium [78,79]. Gold nanoparticles in particular have been used to produce a biocompatible and conductive chitosan hydrogel scaffold for cardiac tissue engineering, successfully promoting the differentiation of seeded MSCs into cardiac lineages through the exploration of the electrical properties of gold nanoparticles [80]. Although extremely versatile, one of the main disadvantages of metal nanoparticles seems to be their short or long-term cytotoxicity depending on their size and composition, which may hinder their use for CTE purposes [81]. Nevertheless, several studies have demonstrated the biocompatibility of metallic nanoparticles, with great room for improvement through the adoption of novel biosynthesis strategies for their production [82,83]. Additionally, the conductivities of the produced scaffolds should attempt to match the physiological values of the target tissue. Despite the high electrical conductivities described for metallic nanoparticles (e.g., gold—41 × 10^6^ S m^−1^; silver—62.9 × 10^6^ S m^−1^; platinum—9.1 × 10^6^ S m^−1^) [79], as such nanoparticles will be incorporated in low conductivity or non-conductive hydrogel materials, the overall conductivity of the composite will be much lower and closer to the values reported for articular cartilage (≈1.2 S m^−1^) [84,85]. Accordingly, Baei and co-workers have developed a gold nanoparticle–chitosan hydrogel with an electrical conductivity of approximately 0.13 S m^−1^ [80]. The composite hydrogel showed high biocompatibility, supporting the migration and proliferation of encapsulated MSCs for 14 days [80]. In another study, Alarcon and colleagues fabricated composite collagen–silver nanoparticles hydrogels, which were demonstrated to be highly biocompatible both for human skin fibroblasts and keratinocytes [86].

### 3.2. Graphene-Based Materials and Carbon Nanotubes

Both graphene and carbon nanotubes (CNTs) are conductive materials with high tensile strengths that can be employed to reinforce and provide conductivity to hydrogel biomatrices with a broad range of applications (Table 3). Graphene, a one atom thick, two-dimensional sheet of carbon atoms, is produced by the peeling of highly pyrolyzed graphite. Hydrogels containing graphene have been used in many fields, including biomedical applications [98], water treatment [99], and as supercapacitors [100]. These platforms have been shown to be not only highly biocompatible, but also to accelerate stem cell growth and differentiation through molecular interactions [101]. Accordingly, Sayyar and colleagues reported that the addition of graphene to a chitosan polymer matrix resulted in a significant enhancement of the hydrogels mechanical strength. The obtained composite showed excellent biocompatibility, supporting fibroblast attachment and growth [102].

CNTs are cylindrical shaped tubes with nanosized diameters. Due to their high tensile strength, excellent electric and thermal conductivity, their coupling with hydrogel matrices has seen a widespread use in several biomedical applications [103,104,105,106]. For example, magnetically fabricated single-wall CNTs in a chitosan hydrogel exhibited enhanced mechanical properties while also improving scaffold’s cytocompatibility to osteoblasts, suggesting this platform’s promising potential for bone tissue engineering strategies [107]. Additionally, our group has recently shown the excellent biocompatibility of a conductive bioink composed of decellularized ECM (dECM) hydrogels incorporating multi-walled CNTs (MWCNTs). The composite hydrogels were shown to improve the contractile behavior of human iPSCs-derived cardiomyocytes [108]. However, according to other studies, carbon nanotubes can exhibit varying levels of cytotoxicity, which are dependent on their purity, shape, size, and functionalization. In fact, CNTs with higher length and diameter are less biocompatible because they cannot be fully engulfed by the macrophages, preventing their clearance from the body and inducing inflammation. In addition, fiber-like or clustered CNTs with larger contact areas showed higher toxicity than nano-spheres/cubes with a lower aspect ratio [109].

### 3.3. Conductive Polymers

Conductive polymers are organic polymers with unique mechanical and optical properties. They have characteristics similar to some metals and inorganic semiconductors while maintaining characteristic polymer properties such as facilitated synthesis and functionalization, versatility, and flexibility [114]. In addition, conductive polymers can be processed using several scaffold manufacturing methods such as casting, 3D-fused deposition modeling, electrospinning, and bioprinting [115,116,117]. Electrically conductive conjugated polymers such as polyaniline (PANi) [118], poly(3,4-ethylene dioxythiophene):polystyrene sulfonate (PEDOT:PSS) [119], polythiophene (PT) [120], and polypyrrole (PPy) [121] have been successfully incorporated into hydrogels and applied in various fields (Table 4). PEDOT:PSS and PPy are among the conjugated polymers with higher electrical conductivity and enhanced biocompatibility. Accordingly, Yang and colleagues fabricated a PPy/alginate biocompatible and conductive hydrogel that significantly improved human MSC proliferation and neural differentiation [122]. However, due to its higher electrochemical stability and ease of processing, PEDOT:PSS has been widely explored in tissue engineering strategies both as coating material and mixed with other biomaterials [123,124,125,126]. Our group has recently showed that PEDOT:PSS coated polybenzimidazole nanofibers were able to promote the adhesion and proliferation of MSCs [124]. Moreover, crystallized PEDOT:PSS has been utilized to produce a 3D printable conductive hydrogel for neural tissue engineering. The scaffold exhibited improved electrochemical properties with minimal cytotoxicity, positively influencing cell adhesion, and proliferation. When combined with electrical stimulation, neural differentiation was significantly enhanced [125]. PEDOT:PSS was also used to produce highly porous conductive scaffolds through ice templating method. The porous scaffolds based purely on PEDOT:PSS were shown to significantly promote the mineralization and osteogenic differentiation of MC3T3-E1 pre-osteoblasts [126].

## 4. Electrically Conductive Hydrogels for Articular Cartilage Tissue Engineering

The fabrication of hydrogels with different materials and properties as cartilage-healing constructs is well documented. However, recently there has been an emergence of a few research studies trying to improve cell-material interactions and more closely mimicking the native AC tissue’s biophysical environment by combining these hydrogels with conductive biomaterials. These new research trends were supported by the known electrical properties of cartilage [84,135] and by studies reporting the positive effects of electrical stimulation in enhancing both the chondrogenic differentiation of MSCs [69,136,137] and chondrocyte growth/ECM production in vitro [138], as well as AC defect repair in vivo [68].

The underlying mechanisms through which endogenous or external electrical stimuli influence cell behavior are still poorly described. However, the alteration of cell membrane resting potential by electrical stimuli, which triggers the voltage-gated calcium channels (VGCC) to open, allowing calcium intake by the cells, is one of the dominant responses. The increase in the intracellular calcium levels activates the calcineurin and calmodulin-mediated signaling pathways, which in turn alter the cells’ gene expression profile and induce the production of growth factors involved in chondrogenesis (e.g., TGF-β and BMPs) [139]. By combining electrical stimulation with inhibitors of TGF-β1 and BMP-2, Kwon and colleagues showed that MSC condensation required for chondrogenesis was significantly improved after electrical stimulation, a process that was mediated by TGF-β signaling [69]. In addition, the activation of mitogen-activated protein kinase (MAPK) signaling pathways is another potential mechanism through which electrical stimulation regulates cell behavior [140]. However, despite the need for further research to fully understand the mechanisms involved in AC formation mediated by electrical stimuli, the use of conductive hydrogels to recreate in vitro the native tissue microenvironment appears to be a promising strategy to improve the existing protocols.

When designing conductive hydrogel scaffolds as cartilage substitutes, some parameters are necessary to consider in order to correctly recapitulating the native environment, including the mechanical strength/stiffness of the hydrogel, its conductivity and biocompatibility. Despite literature on this topic being scarce, the existing results are highly promising, highlighting the potential of electrically conductive hydrogels for improved CTE strategies (Table 5).

Zhang et al., produced a poly(vinyl alcohol) (PVA) hydrogel combined with sodium phytate (PANa), which conferred conductivity and excellent mechanical properties to the hydrogel [141]. The mechanical properties of the hydrogel were studied through rheology with a shear-stress sweep test, with conductivity and swelling behavior also being assessed. The composite was able to resist a strain of over 600% before breaking with a tensile strength of over 7 MPa. Furthermore, its elastic resilience was highlighted by a 20-cycle load–unload test at 0–50% strain, showing negligible changes in tensile strength through the loops. The PVA-PANa hydrogel had a conductivity of about 1.65 S m^−1^, which is in the range of native articular cartilage (≈1.2 S m^−1^, value reported for bovine cartilage [84]). Most hydrogels will gradually absorb water and swell when placed in water, which is an important feature when mimicking native AC tissue as it is a highly water dense tissue. However, some hydrogels might absorb too much water and, in some cases, even dissolve, which severely decreases its mechanical properties. The hydrogel presented in this study exhibited anti-swelling properties, with a 50% increase in weight after 7 days but no significant changes during the next 18 days, maintaining its mechanical stability [141].

In a different study, Shen and colleagues investigated the chondrogenic inducing potential of a graphene oxide (GO) containing poly-D,L-lactic acid/polyethylene glycol (PDLLA) nanocomposite hydrogel [142]. As chondroinductive factors, such as TGF-β, have a low long-term stability and challenging effective delivery, this work was performed in the absence of these factors. In terms of cell viability, the nanocomposite GO/PDLLA hydrogel showed no cytotoxicity during a 21-day culture period, with human bone marrow-derived MSCs proliferating normally. The mechanical properties of the gels were also monitored during the incubation period, with the control PDLLA hydrogel retaining only 1% of its original mechanical strength. In contrast, the GO-incorporated scaffolds managed to retain 5% of their original mechanical strength. Compared to the control PDLLA hydrogel alone, when seeded in the GO/PDLLA nanocomposite, human bone marrow-derived MSCs exhibited a 4-fold and 44-fold higher expression of the chondrogenic markers aggrecan and collagen type II, respectively. Additionally, the expression of SOX9, a key transcription factor involved in controlling the production of cartilage ECM components, was also significantly up-regulated. Finally, the GO/PDLLA hydrogel was biodegradable, which is advantageous for CTE strategies since post injection in the damaged site, it would be degraded and cleared in the kidneys without the need for surgery [142].

3D printing allows layer-by-layer development of 3D structures and the fabrication of complex scaffolds in a fast and reproducible manner. Given that this technique enables a precise control over the scaffold structural features, it is a powerful tool for the fabrication of hydrogels that mimic the intricacies of the heterogeneous structure of AC [143]. Distler et al. tried to take advantage of this by fabricating a 3D-printable oxidized alginate–gelatin (ADA-GEL) polypyrrole:polystyrenesulfonate (PPy:PSS) hydrogel for tissue engineering [121]. Different concentrations of PPy lead to distinct mechanical properties of the final scaffold. Despite testing concentrations ranging from 0.1 to 0.4 M, hydrogels with 0.1 M of PPy exhibited the best mechanical properties, with a tensile strength of ≈1.2 MPa at 40% strain, while the control ADA-GEL hydrogel only reached ≈0.2 MPa. As a result, the produced electroconductive scaffolds were found to be suitable for soft-to-hard tissue tensile strength ranges of ≈1–1.5 MPa, which is close to the range of the native cartilage tissue [121]. In terms of conductivity, the hydrogels with 0.1 M PPy again showed the best results, with a conductivity range close to native cartilage at ≈1.0–1.4 S m^−1^. The PPy modification of the hydrogel led to a slightly reduced attachment and proliferation of ATDC5 cells (pre-chondrogenic cell line), which might result from the increased stiffness of the hydrogel. Nevertheless, by 3D printing the scaffold, the authors were able to obtain an enhanced seeding efficiency throughout the hydrogel’s z-direction [121].

## 5. Challenges and Future Perspectives

The promising results observed in the few studies developing electrically conductive hydrogels for AC regeneration highlight its potential as a suitable culture platform for improved CTE strategies. However, further studies regarding this topic need to be conducted in order to fully assess the potential of these systems for cartilage regeneration. A wider array of hydrogel–conductive material combinations needs to be explored, with particular emphasis on the biodegradability of the produced hydrogels if they are to be implemented in vivo. In fact, a major limitation that is hampering a wider clinical application of these electroactive hydrogels in AC and osteochondral regeneration is the uncontrollable, low, or non-biodegradability of the most used conductive materials. However, some recent strategies targeting other tissues have already reported biodegradable conductive hydrogels either by combining conductive materials with known biodegradable polymers [145,146], using new ionic liquid-grafted hydrogels [147], or exploring less known biodegradable 2D nanomaterials (e.g., black phosphorus) with good electrical conductivity as conductive fillers of hydrogel matrices [148]. However, this list of biodegradable options should be increased for meeting the needs of more versatile and specific applications. In addition, optimization of the hydrogels’ conductivity without inducing inflammatory and toxicity responses upon implantation and without losing the appropriate mechanical properties for AC tissue engineering strategies is a highly challenging task that needs to be continuously explored further.

Another challenge limiting the application of conductive hydrogels in regenerative medicine is their low processability by 3D additive manufacturing techniques. In fact, the fabrication of complex structures based on electrically conductive hydrogels using 3D printing/bioprinting techniques is currently still under-investigated [116]. The use of emerging additive manufacturing technologies such as volumetric printing and two-photon polymerization to fabricate complex and gradient cell-laden conductive hydrogel structures should be explored with high potential for introducing major advances in AC and osteochondral tissue engineering.

Furthermore, a deeper understanding of the tissue’s electrical properties is necessary in order to successfully mimic the AC’s native niche and achieve proper regeneration outcomes. Novel methods to determine the in vivo tissues’ conductivity as well as other electrochemical properties may be explored to better guide tissue engineers in the preparation of biomimetic cartilage substitutes. In addition, there is an urgent need to better understand the underlying mechanisms of how electrical stimulation affects the behavior of the different cell types. The main signaling pathways activated by electrical stimuli and controlling cell proliferation and differentiation need to be further studied and understood. Unraveling such aspects will enable us to fully assess the efficacy of electrical stimuli-based tissue engineering strategies [149]. Considering the promising results reported, electrically conductive scaffolds might be the answer to an ever-growing problem, allowing the direct injection of cell-laden hydrogel into a damaged cartilage site, combined with external electrical stimulation to promote the regeneration of the tissue. Moreover, such smart hydrogels can incorporate drugs or growth factors that will be released in a controlled manner upon electrical stimulation [150]. Based on the latest technological advances on the wireless stimulation of conductive hydrogels [151,152], it might be possible that in the future patients with AC and osteochondral defects can be treated more comfortably at home through the use of a wireless device able to promote tissue regeneration through the controlled electrical stimulation of implanted conductive cartilage substitutes.

## 6. Conclusions

CTE has emerged as an exciting alternative to the current ineffective treatments for cartilage damage and degeneration, as in cases of OA. In particular, the development of electrically conductive hydrogels aiming to replace cartilage tissue and promote its regeneration has seen promising results, mainly due to its potential to not only mimic AC’s mechanical and structural properties, but also recreate its innate electrochemical properties.

In summary, this review provides an overview of the most common conductive materials that have been combined with hydrogels for tissue engineering and other biomedical applications. Conductive materials including metal nanoparticles, carbons, and graphene, and conjugated polymers were highlighted, and their advantages and disadvantages were discussed. Moreover, the few studies focused on the fabrication of electrically conductive hydrogels for CTE purposes were presented and discussed in more detail. Across the analyzed research, it was possible to find different composite hydrogels able to recapitulate the mechanical properties and conductivity of native cartilage while also being able to promote the chondrogenic differentiation of the seeded cells. Despite the research on this topic still being in its infancy, the results reported thus far are extremely promising, highlighting the potential of electrically conductive hydrogels to improve the current suboptimal tissue engineering and regenerative medicine strategies in AC and osteochondral defect repair.

## Figures and Tables

**Table 1:** Examples of hydrogels materials used as injectable systems for CTE applications.

Injectable Hydrogel Material	Advantages	Disadvantages	Refs
Heparin	Naturally occurring negatively charged GAG able to interact with ECM proteins/growth factors and influence several cellular processes.	Poor mechanical properties	[52,53]
Collagen	High biocompatibility Biodegradable Promotes cell adhesion Non-immunogenic Biomimetic of native AC (collagen type II)	Poor mechanical stability Slow gelation Rapid degradation	[54,55]
Gelatin	Cost-effective High biocompatibility Biodegradable Promotes cell adhesion Non-immunogenic	Poor mechanical properties and stability Rapid degradation	[56]
Alginate	Fast gelation Cost-effective Non-immunogenic Non-toxic	Lack of strength to maintain structural shape of the tissue Poor cell attachment	[57]
Poly (ethylene glycol) (PEG)	Adjustable mechanical and structural properties Biocompatibility	Possible immunogenicity Non-biodegradable Poor cell adhesion and growth	[58,59]
Chondroitin sulfate	Easily available High biocompatibility Biodegradable Anti-inflammatory Biomimetic of native AC	Difficult processability Poor mechanical properties	[60]
Hyaluronic Acid	High biocompatibility Biodegradable Promotes cell growth and differentiation Non-immunogenic	Poor mechanical strength Rapid degradation	[61,62]
Chitosan	Biocompatibility Antibacterial and antifungal activity	Poor mechanical properties Poor structural control Extensive swelling in water	[63,64]

**Table 2:** Properties and applications of metallic nanoparticles.

Nanoparticle Material	Advantages	Disadvantages	Applications	References
Gold nanoparticles (Au NPs)	Low initial cytotoxicity High stability	Weak optical signal Long term cytotoxicity High cost	Photodynamic therapy X-ray imaging Drug delivery Cancer treatment	[80,87,88,89]
Silver nanoparticles (Ag NPs)	High optical signal Anti-bacterial and fungal properties	Low stability Cytotoxicity High cost	Cancer treatment Skin and Bone TE Drug delivery	[86,90]
Platinum Nanoparticles (Pt NPs)	High optical signal High stability	High cost Cytotoxicity	Bioimaging Drug delivery Cancer treatment	[91,92,93]
Zinc oxide nanoparticles (ZnO NPs)	High optical signal Economical Anti-bacterial effect Piezoelectric	Low stability Cytotoxicity Require a toxic solvent	Bioimaging Atopic dermatitis treatment Diabetes treatment Cancer treatment	[94,95,96,97]

**Table 3:** Properties and applications of graphene and carbon nanotubes.

Carbon Type	Advantages	Disadvantages	Applications	References
Graphene	High mechanical strength Easily synthesized High conductivity	Oxidative stress Aggregation Possible cytotoxicity	Drug delivery Cancer treatment Tissue engineering Bioimaging	[98,99,100,101,102,110,111,112,113]
Carbon nanotubes (CNTs)	High mechanical strength High conductivity	Oxidative stress Possible cytotoxicity	Tissue engineering Biosensors Drug delivery	[103,104,105,106,107,108]

**Table 4:** Properties and applications of main conductive polymers.

Conductive Polymer Type	Advantages	Disadvantages	Applications	References
Polyaniline (PANi)	High stability High conductivity	Low cell adhesion and growth	Antimicrobial therapy Drug delivery Tissue engineering	[118,127,128,129]
Poly(3,4-ethylene dioxythiophene):polystyrene sulfonate (PEDOT:PSS)	High stability High conductivity Biocompatibility	Low mechanical Strength	Drug delivery Tissue engineering	[119,125,126,130]
Polythiophene (PT)	Good optical properties Biocompatibility	Low conductivity Low stability	Biosensors Tissue engineering	[131,132]
Polypyrrole (PPy)	High conductivity Biocompatibility High mechanical strength	Need for toxic solvent Difficult processability	Drug delivery Tissue engineering Cancer treatment	[122,133,134]

**Table 5:** Summary of research studies on electrically conductive hydrogels for AC tissue engineering.

Hydrogel	Conductive Filler	Main Outcomes	References
Poly(vinyl alcohol) (PVA)	Sodium phytate (PANa)	Easy to produce and cost-effective PVA-PANa hydrogel. Excellent mechanical strength with a fracture stress of over 7 MPa and stable in different solutions for over 20 days. Ionic conductivity of 1.65 S m^−1^. Hydrogel features are close to the properties of native AC.	[141]
Poly-D,L-lactic acid/polyethylene glycol (PDLLA)	Graphene Oxide (GO)	Biodegradable PDLLA-GO nanocomposite hydrogel that promotes hBMSCs chondrogenic differentiation even in the absence of chondroinductive factors. The addition of GO also improved the mechanical properties of the hydrogel.	[142]
Oxidized alginate–gelatin (ADA-GEL)	Polypyrrole: polystyrenesulfonate (PPy:PSS)	Cytocompatible, 3D-printable and electroactive oxidized alginate–gelatin PPy hydrogel that allow improved cell-material interactions. Both the tensile strength (≈1.2 MPa) and conductivity (≈1.0–1.4 S m^−1^) of this hydrogels are within the range of values found in native articular cartilage.	[121]
Chitosan- β-glycerophosphate (CS-ΒGP)	Oligopyyrole (OPy)	Biodegradable and cytocompatible CS-BGP-OPy hydrogel. The addition of OPy significantly increased the conductivity of the scaffold to 1.9 S m^−1^, which is relatively close to the value reported for native cartilage.	[144]

## Data Availability

Not applicable.
